# Using cryo-electron microscopy maps for X-ray structure determination

**DOI:** 10.1107/S2052252518005857

**Published:** 2018-05-11

**Authors:** Lingxiao Zeng, Wei Ding, Quan Hao

**Affiliations:** a School of Biomedical Sciences, University of Hong Kong, 21 Sassoon Road, Hong Kong; b Institute of Physics, Chinese Academy of Sciences, Beijing 100190, People’s Republic of China

**Keywords:** *FSEARCH*, *IPCAS*, iterative phasing, cryo-EM, model building, structure determination, cryo-electron microscopy, X-ray crystallography, phase problem

## Abstract

X-ray crystallography and cryo-electron microscopy (cryo-EM) are complementary techniques for structure determination. Crystallography usually reveals more detailed information, while cryo-EM is an extremely useful technique for studying large-sized macromolecules. As the gap between the resolution of crystallography and cryo-EM data narrows, the cryo-EM map of a macromolecule could serve as an initial model to solve the phase problem of crystal diffraction for high-resolution structure determination. *FSEARCH* is a procedure to utilize the low-resolution molecular shape for crystallographic phasing. The *IPCAS* (Iterative Protein Crystal structure Automatic Solution) pipeline is an automatic direct-methods-aided dual-space iterative phasing and model-building procedure. When only an electron-density map is available as the starting point, *IPCAS* is capable of generating a completed model from the phases of the input map automatically, without the requirement of an initial model. In this study, a hybrid method integrating X-ray crystallography with cryo-EM to help with structure determination is presented. With a cryo-EM map as the starting point, the workflow of the method involves three steps. (1) Cryo-EM map replacement: *FSEARCH* is utilized to find the correct translation and orientation of the cryo-EM map in the crystallographic unit cell and generates the initial low-resolution map. (2) Phase extension: the phases calculated from the correctly placed cryo-EM map are extended to high-resolution X-ray data by non-crystallographic symmetry averaging with *phenix.resolve*. (3) Model building: *IPCAS* is used to generate an initial model using the phase-extended map and perform model completion by iteration. Four cases (the lowest cryo-EM map resolution being 6.9 Å) have been tested for the general applicability of the hybrid method, and almost complete models have been generated for all test cases with reasonable *R*_work_/*R*_free_. The hybrid method therefore provides an automated tool for X-ray structure determination using a cryo-EM map as the starting point.

## Abstract 2

A hybrid method is presented that provides an automated tool for X-ray structure determination using a cryo-EM map as the starting point.

## 1. Introduction   

X-ray crystallography has played a fundamental role in the field of structural biology to provide a mechanistic understanding of critical biological processes. It is a dominant technique for solving molecular structures at atomic or nearly atomic resolution, which allows interpretation of the mechanisms that underlie the biological process; however, producing well ordered three-dimensional crystals is a major bottleneck for large assemblies of multiple components. In recent years, cryo-EM has emerged as a complementary technique using molecules in solution, which opens up the possibility of determining the three-dimensional structures of large molecular complexes and of systems that exhibit multiple conformational or compositional states (Cheng, 2015 ▸). Less than a decade ago, the resolution of images was rarely better than 10 Å, owing to the technical limitations imposed by the instrument, but a revolution occurred around 2012, and since then cryo-EM has experienced dramatic technical advancements such as new electron detectors, phase plate devices and beam-induced motion correction (Li *et al.*, 2013 ▸; Schröder, 2015 ▸; Vénien-Bryan *et al.*, 2017 ▸). These tools have allowed the determination of atomic resolution structures better than 4 Å resolution (Doerr, 2015 ▸).

The different principles of cryo-EM and X-ray crystallography, from specimen preparation to data processing, can complement each other in several ways (Wang & Wang, 2017 ▸). The phase problem arises for crystallographic structure determination because only precise amplitudes are measurable and the phases are lost in diffraction experiments. In the past few decades, several methods have been developed to solve the phase problem. If extremely high-resolution X-ray data are available, one such method is *ab initio* phasing, as implemented in *ARCIMBOLDO* (Rodríguez *et al.*, 2009 ▸). Initial phases can also be derived experimentally from isomorphous or anomalous differences using heavy-atom diffraction, or phases can be obtained using molecular replacement (MR) when suitable models for placement in the unit cell are known. Usually a homologous protein is used as the search model, but as the gap between the resolution of crystallographic and cryo-EM data narrows, using a cryo-EM map of low resolution to help with X-ray structure determination becomes possible. A low-resolution cryo-EM map of an entire molecule provides the overall shape of the molecule, whose sub-components, or their homologues, may be solved by X-ray crystallography. The cryo-EM map of the macromolecule at a reasonable resolution may serve as an initial model to solve the crystallographic phase problem for high-resolution structure determination (Jackson *et al.*, 2014 ▸, Song *et al.*, 2015 ▸). Generally, the procedure can be divided into three parts: cryo-EM map replacement, phase extension and model building.

When the cryo-EM map is correctly placed in the unit cell, the phases are calculated up to the resolution of the cryo-EM map. Xiong has discussed the issues relating to the use of cryo-EM maps as search models for MR using various standard MR packages (*Phaser, AmoRe*, *MOLREP*) (Xiong, 2008 ▸). He proposed several steps that should be carefully dealt with in the process, such as making sure the cryo-EM magnification factor is correct and placing the cryo-EM map into a large *P*1 cell to ensure fine sampling of structure factors. Jackson and co-authors have presented a detailed protocol to explain how a cryo-EM map could be prepared for conventional MR (Jackson *et al.*, 2015 ▸).

We previously developed a procedure named *FSEARCH* that could utilize the low-resolution molecular shape for crystallographic phasing (Hao, 2006 ▸). The source of the envelope can be determined by small-angle X-ray scattering of a solution (SAXS) or cryo-EM. *FSEARCH* has also proved to be powerful in utilizing the molecular envelope of NMR structures as the search model for phasing where conventional MR procedures were unsuccessful (Zhang *et al.*, 2014 ▸). *FSEARCH* simultaneously performs a six-dimensional search on orientation and translation to find the best match between the observed and calculated structure factors. This offers a new choice when conventional MR programs fail to yield a correct solution.

*IPCAS* (Iterative Protein Crystal structure Automatic Solution) is a direct-methods-aided dual-space iterative phasing and model-building pipeline (Zhang, Wu *et al.*, 2010 ▸). In 2015, we demonstrated that starting with a partial model that is as low as 30% of the protein complex, *IPCAS* is capable of extending the starting structure generated from MR to an almost complete complex structure with reasonable *R*_work_ and *R*_free_ values (Zhang *et al.*, 2015 ▸). This procedure integrates several programs and can call these individual programs in three parts of its workflow: (1) reciprocal-space phase refinement by *OASIS* (Zhang, Gu, *et al.*, 2010 ▸); (2) density modification by *DM* (Winn *et al.*, 2011 ▸) or *RESOLVE* (Terwilliger, 2000 ▸); (3) real-space model building and refinement, including *ARP/wARP* (Langer *et al.*, 2008 ▸), *Buccaneer* (Cowtan, 2006 ▸), *Phenix.AutoBuild*, *RESOLVE* (Terwilliger *et al.*, 2008 ▸) and *REFMAC*5 (Murshudov *et al.*, 2011 ▸). The whole procedure can be performed iteratively: during each iterative cycle, a number (from one onwards) of trials run in parallel, and the result from the trial with the highest map-model CC or smallest *R* factor will be passed on to the next cycle until a satisfactory model is obtained. *IPCAS* has been shown to have an advantage particularly for cases where only a small part/subunit is known compared with other widely used model building approaches (Zhang *et al.*, 2015 ▸).

**Figure 1:**
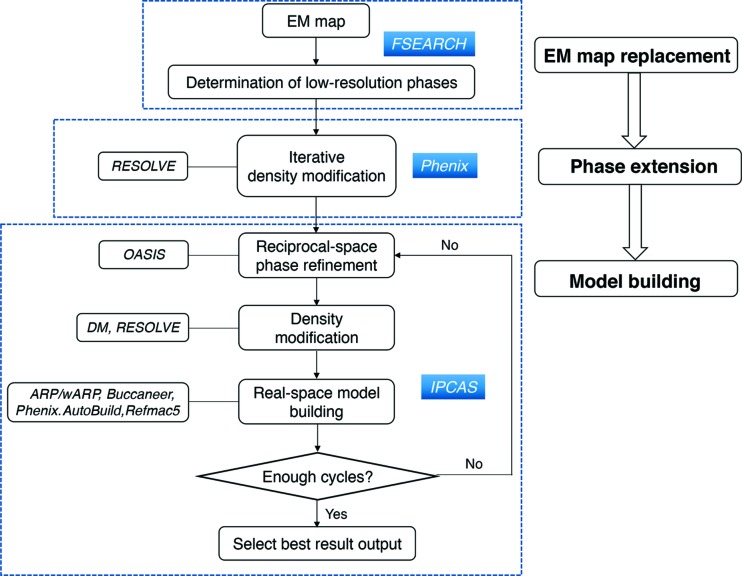
Workflow of a hybrid method integrating X-ray crystallography with cryo-EM for structure determination.

Here we present a hybrid method integrating X-ray crystallography with cryo-EM for structure determination (Fig. 1 ▸). With a cryo-EM map as the starting point, the workflow of the method involves three steps. (1) Cryo-EM map replacement: *FSEARCH* is utilized to find the correct translation and orientation of the cryo-EM map in the crystallographic unit cell and generates the initial low-resolution map. (2) Phase extension: the phases calculated from the correctly placed cryo-EM map are extended to the high-resolution X-ray data by non-crystallographic symmetry (NCS) averaging using *phenix.resolve*. (3) Model building: *IPCAS* is used to generate an initial model using the phase-extended map and perform model completion by iteration.

## 2. Methods   

### 2.1. Map preparation   

If a component of a cryo-EM map is exactly the same as the target structure of X-ray crystallography, the cryo-EM map of an entire molecule can be used directly to provide the overall shape of the target molecule. If a sub-component of a cryo-EM map is the target structure of X-ray crystallography, the map needs to be prepared first. The *Segment Map* tool in *Chimera* (Pintilie *et al.*, 2010 ▸) is part of the *Segger* package which performs watershed segmentation: a density map is partitioned so that each local maximum has its own region, and the boundaries between the regions lie at the valleys between the local maxima. The cryo-EM map is segmented into several regions, then the specified region corresponding to the target molecule is cut out as an input search model.

### 2.2. Cryo-EM map replacement   

The prepared cryo-EM map is delivered to *FSEARCH* (development version) to locate the correct position in the unit cell. The *R* factor is used by the program to evaluate the agreement of calculated and observed structure factors. The correlation coefficient is another indicator of a correct solution and is also applied as a filter to solve any false-positive problems. The *FSEARCH* results are given as a list of translations and orientations sorted in ascending order by *R* factor. A global search is performed to find the best solution, divided into two parts. (i) An initial coarse search: 3–5° steps on Eulerian angles, α, β and γ, and 2–3 Å steps on *x*, *y* and *z*. (ii) A finer search based on the best initial coarse search solutions: 1° steps on α, β and γ, and 1 Å steps on *x*, *y* and *z*. To save computational time, the entire *FSEARCH* execution is split into several small tasks based on Eulerian angle ranges as specified by the user. The split jobs will be assigned to each CPU by the operating system. After the global minimum *R* factor is determined, which indicates the cryo-EM map is correctly positioned in the unit cell, calculated phases up to the EM data resolution can be obtained.

### 2.3. Phase extension   

The initial phases from the map replacement are extended to high resolution X-ray diffraction data by iterated density modification implemented in the program *Phenix* v.1.12-2829 (the *RESOLVE density modification* subroutine) (Terwilliger, 2002 ▸). This strategy is rather powerful when there is a high degree of internal symmetry or sufficient resolution overlap between the X-ray and EM data. It is also possible to extend the map to the highest resolution directly, which often results in a good quality electron-density map for interpretation. However, when the number of NCS copies is low and the resolution gap between cryo-EM and X-ray crystallography data is large, it is necessary to truncate the resolution for phase extension and only extend the phases to an intermediate resolution (as shown in case study 3 below).

### 2.4. Model building   

The electron-density map produced by phase extension is delivered to *IPCAS*. The input data are first passed through a model-building and refinement process implemented in *Phenix.AutoBuild* (quick mode) to derive an initial model, and the resultant model is used as the starting point for direct-methods-aided model completion by iteration, including real-space refinement, direct-methods-aided reciprocal-space refinement and model building, with sequence, solvent content and NCS information assigned. The iteration control in *IPCAS* is set as *OASIS–DM–AutoBuild (quick mode)/Buccaneer* for all test cases. To assess the performance of the combined model-completion approach against a stand-alone automated model-building program, *Phenix.AutoBuild* (in *Phenix* v.1.12-2829) and *Buccaneer* (in *CCP*4 v.7.0.042) are also tested using an individual GUI-based version with default parameters. This is not done to prove which is stronger than the other because both are widely used model-building software packages and have a number of examples of their success. In addition, *Phenix.AutoBuild* contains an iterative density modification/building/refinement procedure while *Buccaneer* may just build a model, so it is unfair to compare them in the same way. In this case, we list their results together in the final table to show that for the current examples, *Phenix.AutoBuild* and *Buccaneer* can yield better results in the *IPCAS* iterations than running the programs individually.

## 3. Results   

The general applicability of the hybrid method has been tested with four case studies in which cryo-EM maps of APC3–APC16 complex, human 26S proteasome, yeast 20S proteasome, and Toll-like receptor 13 were used to solve their X-ray crystal structures. Information about cryo-EM data and X-ray diffraction data is summarized in Table 1 ▸.

### 3.1. Case study 1: APC3–APC16 complex   

**Figure 2:**
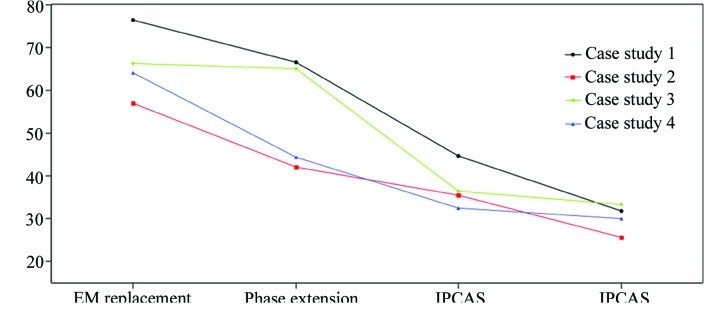
Plot of figure-of-merit-weighted mean phase error (FOM-wMPE) calculated against the crystal structure at the key steps of the whole process.

**Figure 3:**
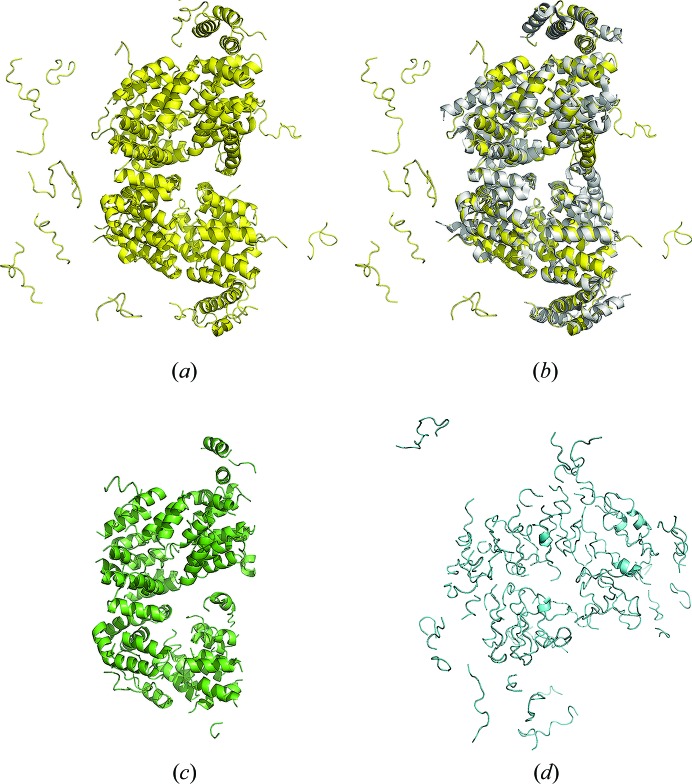
Method comparison, case study 1. (*a*) Model from *IPCAS*; (*b*) model from *IPCAS* superimposed with the crystal structure (PDB entry 4rg6); (*c*) model from *Phenix.AutoBuild*; (*d*) model from *Buccaneer*.

This case was chosen as an example of a small component of the EM map being used to phase the X-ray crystal structure. The anaphase-promoting complex/cyclo­some (APC/C) is a massive E3 ligase that controls mitosis by catalyzing ubiquitination of key cell-cycle regulatory proteins. Within the APC/C complex, APC3 serves as a center for regulation. A part of the cryo-EM map of the APC/C–MCC complex at 4.2 Å resolution (EMDB ID 4037; Alfieri *et al.*, 2016 ▸) was used as the search model for molecular replacement with the X-ray data from the APC3–APC16 complex (PDB entry 4rg6; Yamaguchi *et al.*, 2015 ▸). The initial map was segmented by the *Segment Map* tool in *Chimera* and the part of the map corresponding to the target model was cut out and delivered to *FSEARCH*. For space group *P*4_3_, a five-dimensional envelope search with a fixed *z* position within the unit cell produced a clear solution using crystallographic data (∞–4.2 Å): α = 104, β = 61, γ = 296°, *x* = 3, *y* = 51, *z* = 0 Å. Details are shown in Table 2 ▸. Phases were then calculated from this solution. Since the initial phases are likely to be poor, a model with dummy atoms was generated by *FSEARCH* to produce a mask for helping phase extension. After several cycles of iterative density modification, including solvent flattening, histogram matching, and twofold NCS averaging phases were extended to 3.3 Å. The final phase-extended map was then delivered to *IPCAS* for extension. After the first five cycles (*IPCAS* iteration control: *OASIS*–*DM*–*Phenix.autobuild*), the figure-of-merit (FOM) weighted mean phase error dropped to ∼45° (Fig. 2 ▸). Ten cycles of *OASIS*–*DM*–*Buccaneer* were then performed and the mean phase error in the best cycle dropped to ∼32° (Fig. 2 ▸). Eventually, *IPCAS* could build about 98% of the sequence after 15 cycles of iteration and yield a final model with acceptable refinement statistics (*R*_work_/*R*_free_ = 23.5%/31.9%). The final structure of the APC3–APC16 complex at 3.3 Å resolution determined by *IPCAS* agrees well with previously solved X-ray structures, with an r.m.s.d. (root-mean-square deviation) of 0.535 Å between the *IPCAS* structure and PDB entry 4rg6 (Yamaguchi *et al.*, 2015 ▸). When *OASIS* was disabled during the process (*IPCAS* iteration control: *DM*–*Phenix.autobuild/Buccaneer*), a model was generated with slightly inferior refinement statistics (*R*_work_/*R*_free_ = 26.7%/34.9%). When using stand-alone model building programs, *Phenix.AutoBuild* generated a model with a higher *R*_work_/*R*_free_ compared with the *IPCAS* result, while *Buccaneer* failed to generate a reasonable model (Fig. 3 ▸, Table 3 ▸).

### 3.2. Case study 2: human 26S proteasome   

**Figure 4:**
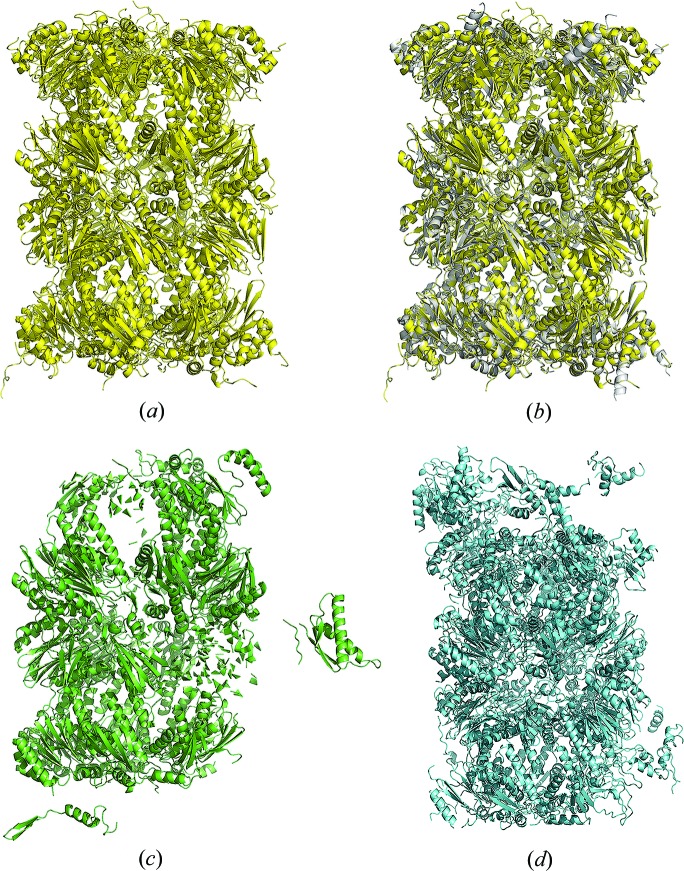
Method comparison, case study 2. (*a*) Model from *IPCAS*; (*b*) model from *IPCAS* superimposed with the crystal structure (PDB entry 5lf7); (*c*) model from *Phenix.AutoBuild*; (*d*) model from *Buccaneer*.

In this case, a major part of the cryo-EM map of the human proteasome bound to the deubiquitinating enzyme USP14 at 4.35 Å resolution (EMDB ID 9511; Huang *et al.*, 2016 ▸) was used as the search model for molecular replacement with the X-ray data from the human 26S proteasome (PDB entry 5lf7; Schrader *et al.*, 2016 ▸). The initial map was segmented by the *Segment Map* tool in *Chimera* and the part of the map corresponding to the target model was cut out and delivered to *FSEARCH*. To save computation time, a self-rotation function with the crystallographic data using *MOLREP* in the *CCP*4 suite (Vagin & Teplyakov, 1997 ▸) yielded two Eulerian angles (α = 78, β = 90°) for the NCS axis of the molecular shape, which reduced the potential six-dimensional search to four dimensions. The search results are listed in Table 2 ▸. The *R* factor of the top solution is 0.570 and there is a clear gap in *R* factors between the top three solutions, which indicates that the search was successful. The top solution was further refined to α = 75, β = 89, γ = 353°, *x* = 8, *y* = 25, *z* = 149 Å. Phase extension by twofold NCS averaging was carried out from a phase calculated from an EM map correctly placed by *FSEARCH*, and resulted in the phases with an FOM weighted mean phase error of ∼42°. After 15 cycles of *OASIS*–*DM*–*Phenix.AutoBuild*/*Buccaneer*, the mean phase error in the best cycle dropped to ∼26° (Fig. 2 ▸). Eventually, *IPCAS* could build about 97% of the sequence after 15 cycles of iteration and yield a final model with acceptable refinement statistics (*R*_work_/*R*_free_ = 23.7%/28.6%). The final structure of the human 26S proteasome at 2.3 Å resolution determined by *IPCAS* agrees well with previously solved X-ray structures, with an r.m.s.d. of 0.455 Å between the *IPCAS* structure and PDB entry 5lf7 (Schrader *et al.*, 2016 ▸). When *OASIS* was disabled during the process (*IPCAS* iteration control: *DM-Phenix.autobuild-Buccaneer*), a model was obtained with acceptable refinement statistics (*R*_work_/*R*_free_ = 23.8%/28.5%). In comparison, both *Phenix.AutoBuild* and *Buccaneer* alone could finish model building but only built about 70% of the sequence (Fig. 4 ▸, Table 3 ▸).

### 3.3. Case study 3: yeast 20S proteasome   

**Figure 5:**
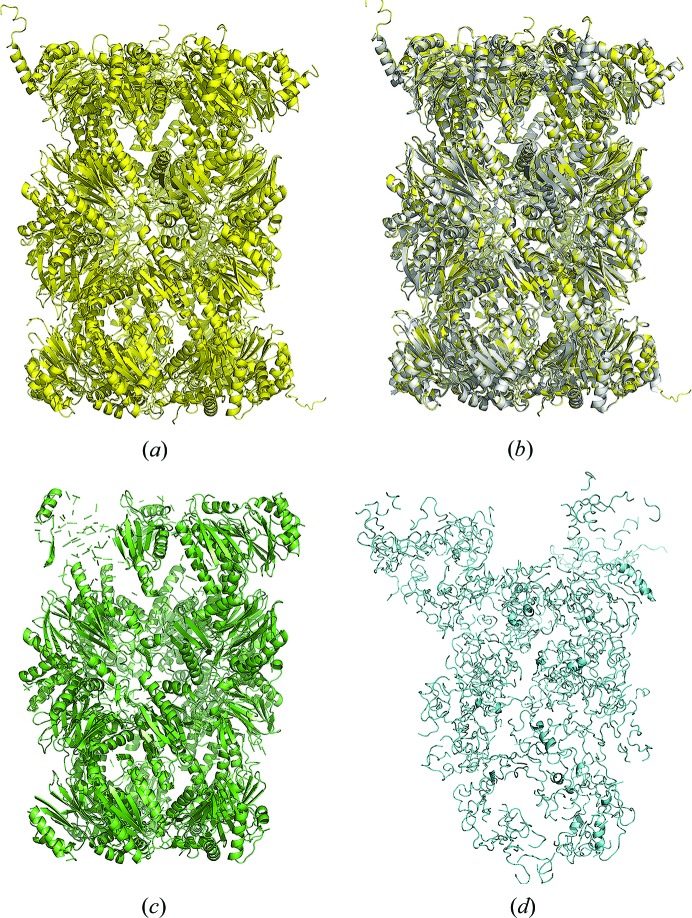
Method comparison, case study 3. (*a*) Model from *IPCAS*; (*b*) model from *IPCAS* superimposed with the crystal structure (PDB entry 5cz4); (*c*) model from *Phenix.AutoBuild*; (*d*) model from *Buccaneer*.

This case represents a large resolution gap between the cryo-EM and X-ray crystallography data. The yeast 20S proteasome is composed of two copies of 14 different subunits (seven distinct α-type and seven distinct β-type subunits) arranged in four stacked rings. In this case, a cryo-EM map at 6.9 Å resolution (EMDB ID 5593; Park *et al.*, 2013 ▸) was used as the search model for molecular replacement with the X-ray data from the yeast 20S proteasome (PDB entry 5cz4; Huber *et al.*, 2016 ▸). For space group *P*2_1_, a five-dimensional envelope search with a fixed *y*-position within the unit cell using crystallographic data (∞–6.9 Å) produced a clear solution: α = 293, β = 5, γ = 70°, *x* = 39, *y* = 0, *z* = 12 Å. Details are shown in Table 2 ▸. Phases were then calculated from this solution. The resolution of cryo-EM map is rather low which brings challenges to phase extension. To overcome the huge gap between the resolution from crystallography and cryo-EM, we therefore truncated the resolution to 3.2 Å yielding an interpretable map for model completion. The final phase-extended map was delivered to *IPCAS*. After the first five cycles (*IPCAS* iteration control: *OASIS*–*DM*–*Phenix.autobuild*), the FOM-weighted mean phase error dropped to ∼36° (Fig. 2 ▸). Ten cycles of *OASIS*–*DM*–*Buccaneer* iteration were then performed and the mean phase error in the best cycle dropped to ∼33° (Fig. 2 ▸). Eventually, *IPCAS* managed to produce a model of 6445 residues (∼97% of the whole structure), all docked into the sequence and yield a final model with acceptable refinement statistics (*R*_work_/*R*_free_ = 21.7%/24.9%). When *OASIS* was disabled during the process (*IPCAS* iteration control: *DM*–*Phenix.autobuild*/*Buccaneer*), a model was obtained with acceptable refinement statistics (*R*_work_/*R*_free_ = 22.4%/26.0%). In comparison, *Phenix.AutoBuild* and *Buccaneer* could also build the final models but the *R*_work_/*R*_free_ (26.7%/30.1% and 51.5%/53.5%, respectively) are not as good as those of the model built by *IPCAS* (Fig. 5 ▸, Table 3 ▸).

### 3.4. Case study 4: Toll-like receptor 13   

**Figure 6:**
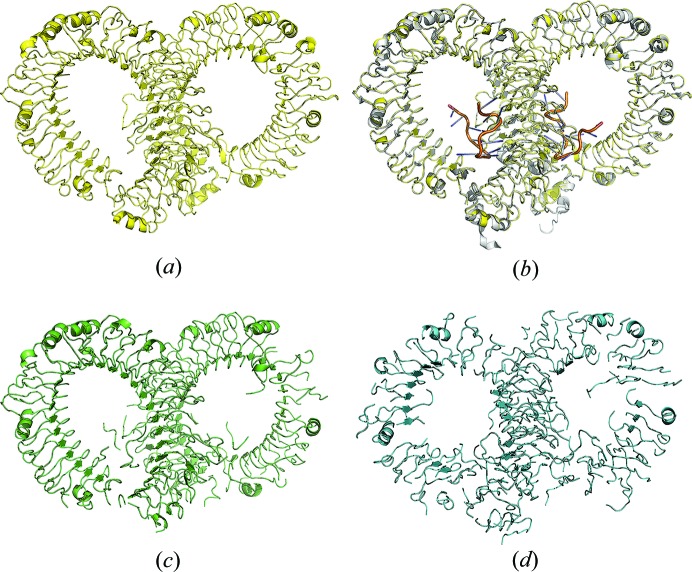
Method comparison, case study 4. (*a*) Model from *IPCAS*; (*b*) model from *IPCAS* superimposed with the crystal structure (PDB entry 4z0c); (*c*) model from *Phenix.AutoBuild*; (*d*) model from *Buccaneer*.

In this case, the original structure determination turned out to be very difficult because of the low number of NCS copies (Song *et al.*, 2015 ▸). We used a cryo-EM map at 4.87 Å resolution (EMDB ID 3125; Song *et al.*, 2015 ▸) as the search model for molecular replacement with the X-ray data from Toll-like receptor 13 (PDB entry 4z0c; Song *et al.*, 2015 ▸). A six-dimensional envelope search was performed within the unit cell using crystallographic data (∞–4.87 Å) and produced a clear solution: α = 329, β = 49, γ = 293°, *x* = 34, *y* = 35, *z* = 73 Å. Details are shown in Table 2 ▸. Phases were then calculated from this solution and extended to 2.3 Å during the phase extension. The final phase-extended map was delivered to *IPCAS*. After the first five cycles (*IPCAS* iteration control: *OASIS*–*DM*–*Phenix.autobuild*), the FOM-weighted mean phase error dropped to ∼32° (Fig. 2 ▸). Ten cycles of *OASIS*–*DM*–*Buccaneer* were then performed and the mean phase error in the best cycle dropped to ∼30° (Fig. 2 ▸). Eventually, *IPCAS* could build about 97% of the sequence after 15 cycles of iteration and yield a final model with acceptable refinement statistics (*R*_work_/*R*_free_ = 27.1%/32.9%). When *OASIS* was disabled during the process (*IPCAS* iteration control: *DM*–*Phenix.autobuild*/*Buccaneer*), a model was obtained with acceptable refinement statistics (*R*_work_/*R*_free_ = 28.0%/34.6%). In comparison, *Phenix.AutoBuild* and *Buccaneer* could also build the final models but the *R*_work_/*R*_free_ (30.5%/35.1% and 48.0%/50.7%, respectively) were not as good as that of the model built by *IPCAS* (Fig. 6 ▸, Table 3 ▸).

## 4. Discussion   

Many case studies have shown that a cryo-EM map could serve as a viable model for molecular replacement in X-ray crystal structure determination, but little has been discussed about automated model completion after MR. Typically, tedious effort is required to manually build a model against the electron-density map. In this study, we have demonstrated a hybrid method that is particularly suitable for model completion when using cryo-EM maps as MR search models.

The use of cryo-EM maps for MR exactly parallels the use of atomic coordinates, the heart of which is a six-dimensional search task. The conventional MR method splits the six-dimensional search task into two sequential three-dimensional search steps using the rotational and translational Patterson functions. These two-step strategies greatly improve efficiency, but in handling low-resolution search models, they may fail in difficult situations when the rotational peaks and translational peaks interfere with each other (Liu *et al.*, 2003 ▸). Also, to be recognized by standard MR packages, a model-preparation step needs to be carried out, which involves the Fourier transform of structure factors and placing the model in a large *P*1 cell with dimensions three or four times as large as those of the model (Xiong, 2008 ▸). In the current study, an alternative method using *FSEARCH* is offered which is not based on a Patterson function but based on *R* factors or correlation coefficients computed from standard or normalized structure factors. *FSEARCH* performs a six-dimensional search using the molecular envelope which is suitable for dealing with low-resolution molecular replacement. It yielded correct solutions in all four cases. The actual CPU time consumed by a four-dimensional, five-dimensional and six-dimensional search on a 16-processor workstation (Intel Xeon Processor E5-1680 v3 @3.2 GHz) was ∼1, 38 and 532 h, respectively.

All cases have only two NCS copies. In the cases of the APC3–APC16 complex, the human 20S proteasome and the Toll-like receptor 13, the resolution gap between cryo-EM and X-ray crystallography is small. After obtaining an MR solution, it is possible to perform phase extension directly on the highest resolution X-ray diffraction data, which results in a reasonable initial electron-density map. It is worth noting that in the case of the yeast 20S proteasome, the resolution gap between cryo-EM and X-ray crystallography data is large and it is therefore necessary to truncate the resolution to an intermediate resolution for phase extension (3.2 Å in this case) in order to obtain an interpretable electron-density map, and all reflections are used in the next phase-improvement/model-building stage.

Test cases have demonstrated that the partial models generated from the phase-extended map could be extended almost to completion for all four cases. The actual CPU time consumed for a moderate-sized (100–200 kDa) protein (such as case studies 1 and 4) by *IPCAS* was about 50–144 h for 15 cycles (each includes three trials); for a large-sized (∼700 kDa) protein (such as case studies 2 and 3), the actual CPU time consumed by *IPCAS* was about 264 h for 15 cycles (each includes three trials). The combination of *OASIS* with the density-modification program (*DM*) and model-building programs (*Phenix.AutoBuild* and *Buccaneer*) leads to dual-space phase improvement which dramatically decreases the phase error, resulting in significant improvements in both the accuracy and completeness of the model compared with the stand-alone model-building programs. This suggests that *IPCAS* alleviates the dependence on satisfactory starting phases. We expect that this hybrid method may provide an option for challenging cases where a homologous structure is unavailable and a cryo-EM map is used for molecular replacement, as well as to improve the efficiency and reliability of the final model completion.

## Figures and Tables

**Table 1:** Cryo-EM and X-ray diffraction data used in the case studies

	Case study 1	Case study 2	Case study 3	Case study 4
Cryo-EM data				
EMDB ID	4037	9511	5593	3125
Resolution (Å)	4.2	4.35	6.9	4.87
Reference	Alfieri *et al.* (2016 ▸)	Huang *et al.* (2016 ▸)	Park *et al.* (2013 ▸)	Song *et al.* (2015 ▸)
				
X-ray diffraction data				
PDB entry	4rg6	5lf7	5cz4	4z0c
Resolution (Å)	3.3	2.0	2.3	2.3
Space group	*P*4_3_	*P*2_1_2_1_2_1_	*P*2_1_	*P*2_1_2_1_2_1_
Unit-cell parameters				
*a*, *b*, *c* (Å)	116.7, 116.7, 185.0	113.4, 202.6, 314.5	137.0, 300.9, 145.8	112.6, 115.2, 167.6
α, β, γ (°)	90, 90, 90	90, 90, 90	90, 113.1, 90	90, 90, 90
Protein residues	1163	6458	6614	1444
NCS copies	2	2	2	2
Reference	Yamaguchi *et al.* (2015 ▸)	Schrader *et al.* (2016 ▸)	Huber *et al.* (2016 ▸)	Song *et al.* (2015 ▸)

**Table 2:** Molecular-replacement solutions for four test cases The results are given in ascending order of *R* factor for the top three solutions. The top solution was chosen to place the map in the unit cell and was output to .mtz files.

	α (°)	β (°)	γ (°)	*x *(Å)	*y *(Å)	*z* (Å)	*R* factor	Correlation coefficient	Starting FOM
Case study 1									
Coarse search	105	60	295	4	50	0	0.549	0.504	
	100	60	300	2	50	0	0.564	0.478	
	105	65	295	2	52	0	0.569	0.458	
Finer search	104	61	296	3	51	0	0.539	0.524	0.484
									
Case study 2									
Coarse search	78	90	354	15	27	150	0.570	0.364	
	78	90	351	9	30	153	0.589	0.307	
	78	90	354	12	24	150	0.606	0.263	
Finer search	75	89	353	8	25	149	0.528	0.465	0.540
									
Case study 3									
Coarse search	280	5	85	48	0	8	0.548	0.232	
	290	5	75	44	0	10	0.551	0.224	
	285	5	80	46	0	10	0.557	0.211	
Finer search	293	5	70	39	0	12	0.519	0.303	0.587
									
Case study 4									
Coarse search	325	50	295	40	38	76	0.595	0.300	
	330	50	290	38	38	74	0.606	0.272	
	330	50	295	30	30	78	0.609	0.261	
Finer search	329	49	293	34	35	73	0.565	0.364	0.532

**Table 3:** Phase-extension and model-completion results The numbers in parentheses for the number of residues built are given as percentages.

	Case study 1	Case study 2	Case study 3	Case study 4
*IPCAS*				
No. of residues built	1116 (96)	6283 (97)	6445 (97)	1406 (97)
*R*_work_/*R*_free_ (%)	23.5/31.9	23.7/28.6	21.7/24.9	27.1/32.9
*IPCAS* (without *OASIS*)				
No. of residues built	1118 (96)	6312 (98)	6521 (99)	1418 (98)
*R*_work_/*R*_free_ (%)	26.7/34.9	23.8/28.5	22.4/26.0	28.0/34.6
*Phenix.AutoBuild*				
No. of residues built	703 (60)	4614 (71)	5229 (79)	1309 (91)
*R*_work_/*R*_free_ (%)	29.6/33.7	29.8/33.6	26.7/30.1	30.5/35.1
*Buccaneer*				
No. of residues built	706 (61)	5094 (79)	3136 (47)	1208 (84)
*R*_work_/*R*_free_ (%)	48.1/50.8	38.9/42.7	51.5/53.5	48.0/50.7

## References

[bb1] Alfieri, C., Chang, L., Zhang, Z., Yang, J., Maslen, S., Skehel, M. & Barford, D. (2016). *Nature*, **536**, 431–436. 10.1038/nature19083 PMC5019344 27509861

[bb2] Cheng, Y. (2015). *Cell*, **161**, 450–457. 10.1016/j.cell.2015.03.049 PMC4409662 25910205

[bb3] Cowtan, K. (2006). *Acta Cryst.* D**62**, 1002–1011. 10.1107/S0907444906022116 16929101

[bb4] Doerr, A. (2015). *Nat. Methods*, **12**, 598–599. 10.1038/nmeth.3469 26339705

[bb5] Hao, Q. (2006). *Acta Cryst.* D**62**, 909–914. 10.1107/S0907444906014089 16855308

[bb6] Huang, X., Luan, B., Wu, J. & Shi, Y. (2016). *Nat. Struct. Mol. Biol.* **23**, 778–785. 10.1038/nsmb.3273 27428775

[bb7] Huber, E. M., Heinemeyer, W., Li, X., Arendt, C. S., Hochstrasser, M. & Groll, M. (2016). *Nat. Commun.* **7**, 10900. 10.1038/ncomms10900 PMC4792962 26964885

[bb8] Jackson, R. N., Golden, S. M., van Erp, P. B., Carter, J., Westra, E. R., Brouns, S. J., van der Oost, J., Terwilliger, T. C., Read, R. J. & Wiedenheft, B. (2014). *Science*, **345**, 1473–1479. 10.1126/science.1256328 PMC4188430 25103409

[bb9] Jackson, R. N., McCoy, A. J., Terwilliger, T. C., Read, R. J. & Wiedenheft, B. (2015). *Nat. Protoc.* **10**, 1275–1284. 10.1038/nprot.2015.069 PMC4620705 26226459

[bb10] Langer, G., Cohen, S. X., Lamzin, V. S. & Perrakis, A. (2008). *Nat. Protoc.* **3**, 1171–1179. 10.1038/nprot.2008.91 PMC2582149 18600222

[bb11] Li, X., Mooney, P., Zheng, S., Booth, C. R., Braunfeld, M. B., Gubbens, S., Agard, D. A. & Cheng, Y. (2013). *Nat. Methods*, **10**, 584–590. 10.1038/nmeth.2472 PMC3684049 23644547

[bb12] Liu, Q., Weaver, A. J., Xiang, T., Thiel, D. J. & Hao, Q. (2003). *Acta Cryst.* D**59**, 1016–1019. 10.1107/s090744490300739x 12777764

[bb13] Murshudov, G. N., Skubák, P., Lebedev, A. A., Pannu, N. S., Steiner, R. A., Nicholls, R. A., Winn, M. D., Long, F. & Vagin, A. A. (2011). *Acta Cryst.* D**67**, 355–367. 10.1107/S0907444911001314 PMC3069751 21460454

[bb14] Park, S., Li, X. M., Kim, H. M., Singh, C. R., Tian, G., Hoyt, M. A., Lovell, S., Battaile, K. P., Zolkiewski, M., Coffino, P., Roelofs, J., Cheng, Y. F. & Finley, D. (2013). *Nature*, **497**, 512–516. 10.1038/nature12123 PMC3687086 23644457

[bb15] Pintilie, G. D., Zhang, J., Goddard, T. D., Chiu, W. & Gossard, D. C. (2010). *J. Struct. Biol.* **170**, 427–438. 10.1016/j.jsb.2010.03.007 PMC2874196 20338243

[bb16] Rodríguez, D. D., Grosse, C., Himmel, S., González, C., de Ilarduya, I. M., Becker, S., Sheldrick, G. M. & Usón, I. (2009). *Nat. Methods*, **6**, 651–653. 10.1038/nmeth.1365 19684596

[bb17] Schrader, J., Henneberg, F., Mata, R. A., Tittmann, K., Schneider, T. R., Stark, H., Bourenkov, G. & Chari, A. (2016). *Science*, **353**, 594–598. 10.1126/science.aaf8993 27493187

[bb18] Schröder, R. R. (2015). *Arch. Biochem. Biophys.* **581**, 25–38. 10.1016/j.abb.2015.05.010 26032338

[bb19] Song, W., Wang, J., Han, Z. F., Zhang, Y. F., Zhang, H. Q., Wang, W. G., Chang, J. B., Xia, B. S., Fan, S. L., Zhang, D. K., Wang, J. W., Wang, H. W. & Chai, J. J. (2015). *Nat. Struct. Mol. Biol.* **22**, 782–787. 10.1038/nsmb.3080 26323037

[bb20] Terwilliger, T. C. (2000). *Acta Cryst.* D**56**, 965–972. 10.1107/S0907444900005072 PMC2792768 10944333

[bb21] Terwilliger, T. C. (2002). *Acta Cryst.* D**58**, 2082–2086. 10.1107/S0907444902016360 PMC2745884 12454468

[bb22] Terwilliger, T. C., Grosse-Kunstleve, R. W., Afonine, P. V., Moriarty, N. W., Zwart, P. H., Hung, L.-W., Read, R. J. & Adams, P. D. (2008). *Acta Cryst.* D**64**, 61–69. 10.1107/S090744490705024X PMC2394820 18094468

[bb23] Vagin, A. & Teplyakov, A. (1997). *J. Appl. Cryst.* **30**, 1022–1025.

[bb24] Vénien-Bryan, C., Li, Z., Vuillard, L. & Boutin, J. A. (2017). *Acta Cryst.* F**73**, 174–183. 10.1107/S2053230X17003740 PMC5379166 28368275

[bb25] Wang, H. W. & Wang, J. W. (2017). *Protein Sci.* **26**, 32–39. 10.1002/pro.3022 PMC5192981 27543495

[bb26] Winn, M. D., Ballard, C. C., Cowtan, K. D., Dodson, E. J., Emsley, P., Evans, P. R., Keegan, R. M., Krissinel, E. B., Leslie, A. G. W., McCoy, A., McNicholas, S. J., Murshudov, G. N., Pannu, N. S., Potterton, E. A., Powell, H. R., Read, R. J., Vagin, A. & Wilson, K. S. (2011). *Acta Cryst.* D**67**, 235–242. 10.1107/S0907444910045749 PMC3069738 21460441

[bb27] Xiong, Y. (2008). *Acta Cryst.* D**64**, 76–82. 10.1107/S090744490705398X PMC2394795 18094470

[bb28] Yamaguchi, M., Yu, S., Qiao, R., Weissmann, F., Miller, D. J., VanderLinden, R., Brown, N. G., Frye, J. J., Peters, J. M. & Schulman, B. A. (2015). *J. Mol. Biol.* **427**, 1748–1764. 10.1016/j.jmb.2014.11.020 PMC4444369 25490258

[bb29] Zhang, T., Gu, Y. X., Zheng, C. D. & Fan, H. F. (2010). *Chin. Phys. B*, **19**, 086103.

[bb30] Zhang, T., Wu, L. J., Gu, Y. X., Zheng, C. D. & Fan, H. F. (2010). *Chin. Phys. B*, **19**, 096101.

[bb31] Zhang, W., Zhang, H., Zhang, T., Fan, H. & Hao, Q. (2015). *Acta Cryst.* D**71**, 1487–1492. 10.1107/S1399004715008597 26143920

[bb32] Zhang, W., Zhang, T., Zhang, H. & Hao, Q. (2014). *Acta Cryst.* D**70**, 1977–1982. 10.1107/S1399004714009754 25004974

